# Impact-induced amino acid formation on Hadean Earth and Noachian Mars

**DOI:** 10.1038/s41598-020-66112-8

**Published:** 2020-06-08

**Authors:** Yuto Takeuchi, Yoshihiro Furukawa, Takamichi Kobayashi, Toshimori Sekine, Naoki Terada, Takeshi Kakegawa

**Affiliations:** 1 Department of Earth Science, Tohoku University, 6-3 Aza-aoba, Aramaki, Aoba-ku, Sendai, 980-8578 Japan; 2 National Institute for Materials Science, 1-1 Namiki, Tsukuba, 305-0044 Japan; 3 Center for High Pressure Science & Technology Advanced Research, 1690 Cailun road, Shanghai, 201203 China; 4 Graduate School of Engineering, Osaka University, Osaka, Japan, 2-1 Yamada-Oka, Suita, 565-0871 Japan; 5 Department of Geophysics, Tohoku University, 6-3 Aza-aoba, Aramaki, Aoba-ku, Sendai, 980-8578 Japan

**Keywords:** Origin of life, Astrobiology, Geochemistry

## Abstract

Abiotic synthesis of biomolecules is an essential step for the chemical origin of life. Many attempts have succeeded in synthesizing biomolecules, including amino acids and nucleobases (e.g., via spark discharge, impact shock, and hydrothermal heating), from reduced compounds that may have been limited in their availabilities on Hadean Earth and Noachian Mars. On the other hand, formation of amino-acids and nucleobases from CO_2_ and N_2_ (i.e., the most abundant C and N sources on Earth during the Hadean) has been limited via spark discharge. Here, we demonstrate the synthesis of amino acids by laboratory impact-induced reactions among simple inorganic mixtures: Fe, Ni, Mg_2_SiO_4_, H_2_O, CO_2_, and N_2_, by coupling the reduction of CO_2_, N_2_, and H_2_O with the oxidation of metallic Fe and Ni. These chemical processes simulated the possible reactions at impacts of Fe-bearing meteorites/asteroids on oceans with a CO_2_ and N_2_ atmosphere. The results indicate that hypervelocity impact was a source of amino acids on the Earth during the Hadean and potentially on Mars during the Noachian. Amino acids formed during such events could more readily polymerize in the next step of the chemical evolution, as impact events locally form amino acids at the impact sites.

## Introduction

The composition of early Earth’s atmosphere has been a subject of discussion. The atmosphere was once regarded as strongly reduced, composed mostly of CH_4_, NH_3_, and H_2_^1^. Since approximately 40 years ago, a CO_2_-N_2_ dominated neutral atmosphere which was equilibrated to oxidized silicate mantle/crust, has been favored^2–5^. More recently, addition of reduced species to the atmosphere by the late veneer and the late heavy bombardment (LHB) have been proposed^6–11^. Although such local events might have provided reduced volatiles, the quantity of reduced species provided to the atmosphere remained unclear.

Initiated by Miller’s 1953 experiment, the formation of amino acids and nucleobases by spark discharge from reduced reactants (e.g., CO, CH_4_, and H_2_) has long been investigated as a potential source of the building blocks of life^12,13^. The yields of amino acids and other organic compounds are significantly sensitive to the amounts of reduced compounds^13^. The formation of amino acids from non-reduced species (i.e., CO_2_, N_2_, and H_2_O) by spark discharge has been proposed^14,15^. The formation reported in Plankensteiner *et al*.^14^ is coupled by the oxidation of Cu electrode, which is not available in natural spark discharge in the atmosphere. Spark-discharge with carbonate buffer reported by Cleaves *et al*.^15^ might have worked as a source of prebiotic amino acid, although ascorbate hydrolysis used in the work was challenged by a subsequent spark discharge study as a source of amino acid contamination^16^. Therefore, an experimentally-supported geological event that synthesizes amino acids is limited in spark discharge on neutral ocean. For other methods, such as shock-heating of the atmosphere, photochemical reactions, and proton irradiation, studies have reported that the formation of amino acids were limited to experiments using reduced C and N sources^12,13,17–22^. Formation of nucleobases is more significantly limited to experiments using reduced C and N sources^21,23–25^. They have not, however, been established using CO_2_ and N_2_, which were major terrestrial sources of C and N during the Hadean. A previous work reported amino acid formation by laser irradiation to CO_2_-N_2_-H_2_O without reductants^26^. However, it is not clear whether the detected amino acids were products or contaminants.

Several studies propose a high CO_2_ partial pressure for the early Hadean^2,3,27^. This suggestion leads to the possibility that concentrations of dissolved inorganic carbon, e.g., HCO_3_^−^, in the Hadean oceans were most likely much higher than of those in today’s oceans. Several sources of NH_3_ during the Hadean have been proposed. Most of these previous studies investigated the reduction of nitrogen oxides by Fe-sulfides, metallic Fe, or Fe^2+^ in rocks or ocean^28–31^. Even considering these sources, models suggest that there were significantly low NH_3_ concentrations in the oceans (e.g., 10^−5^ mol/L)^32^. Formation of organic compounds around hydrothermal vents is a popular model for an endogenous organic source. However, the formation of amino acids has only been demonstrated in experiments using fluid analogues containing reduced C and reduced N compounds (e.g., formaldehyde, NH_3_, and HCN) in extremely high concentrations^19,33^. Amino-acid synthesis has not been shown using non-reduced compounds, which were common in hydrothermal fluids^34^. Furthermore, the chemical equilibrium at high temperatures that are characteristic of hydrothermal vents has been found to favor amino-acid degradation rather than synthesis^34^.

Lunar crater records suggest intense impacts of meteorites and asteroids on the Hadean Earth^35^. Hypervelocity impacts of meteorites and asteroids on water generated post-impact vapor plumes and induced reactions between projectiles, water, and the atmosphere^9,36^. Nakazawa *et al*.^37^ and Nakazawa^38^ proposed that post-impact reactions, which were generated by Fe-bearing projectiles, formed organic compounds essential for life. Both experimental and theoretical studies have indicated that impacts of Fe-bearing asteroids and meteorites reduced the surrounding atmosphere and formed a variety of reduced compounds (e.g., CO, NH_3_, and HCN)^8,10,37,39^. Furthermore, other studies have demonstrated the formation of amino acids and nucleobases in impact-induced reactions between Fe-bearing meteorite analogues, H_2_O, NH_3_, HCO_3_^−^ or solid C, and gaseous N_2_^20,21^. These experiments are demonstrations of the synthesis of amino acids using non-reduced carbon sources (i.e., solid C and HCO_3_^−^) and a reduced nitrogen source (i.e., NH_3_). Metallic Fe worked as a reductant and a catalyst in organic synthesis in these experiments. An catalytic effect by metallic Fe-Ni on reactions between organic compounds, on impact, was suggested by a previous work^40^. Among cataloged meteorites, >85% were found to contain significant amounts of metallic Fe^41^. For example, H chondrite, which is the most common type of ordinary chondrite, has been found to contain ~10 vol% of metals^42^. The impact synthesis model is consistent with a model recently proposed by Benner and co-workers in which large single impactor reduced surrounding materials and formed organic compounds^43^.

We conducted laboratory shock-recovery experiments to investigate impact-induced reactions between a meteorite analogue, oceanic components, and the atmosphere. The experimental setup and analytical procedures are described elsewhere^21^. Mixed powders of forsterite (Mg_2_SiO_4_; 200 mg), metallic Fe (100 mg), and metallic Ni (10 mg) were used as an ordinary chondrite (OC) analogue whereas mixed powders of metallic Fe (300 mg) and metallic Ni (10 mg) were used as an iron meteorite (IM) analogue (Table S1). The impact velocity was ~0.9 km/s, which generated a shockwave of ~7 GPa for 0.7 μsec. The temperature during the shock compression was ~300 °C. Post-shock temperature was estimated to be ~1500 °C^44^. NaHCO_3_ was used as the C source. Dissolved HCO_3_^−^ and CO_2_ were generated from NaHCO_3_ before and during the shock-induced reactions, and these represent the C species in the ocean and atmosphere, respectively. Carbon in NaHCO_3_ was labeled using ^13^C to distinguish products from potential contaminants. Gaseous N_2_ was used as a N source to simulate atmospheric N for all experiments. NH_3_ was mixed with the starting materials in several experiments for comparison. ^13^C-labeled amino acids in the products were analyzed by liquid chromatography tandem mass spectrometry.

**Figure 1:**
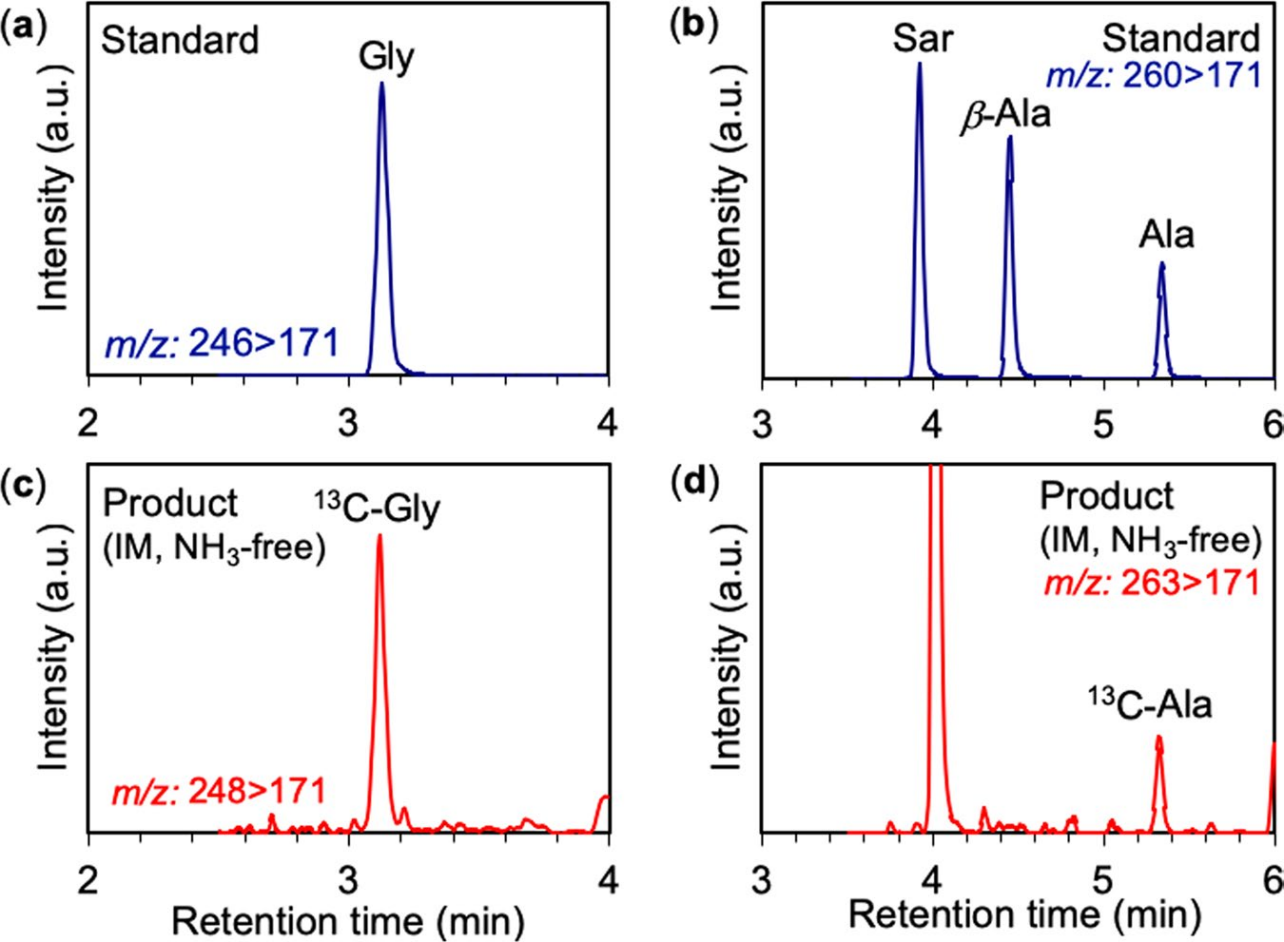
Multiple reaction monitoring (MRM) chromatogram of derivatized standards and the derivatized product of NH_3_-free experiment with the iron meteorite (IM) analogue. (**a**) ^12^C-glycine (Gly) (mass-to-charge ratio (*m/z*) = 246 > 171) and product, ^13^C-glycine (^13^C-Gly) (*m/z* = 248 > 171). (**b**) ^12^C-alanine (Ala), ^12^C-*β*-alanine (*β*-Ala), and sarcosine (Sar) (*m/z* = 260 > 171) and product.

**Figure 2:**
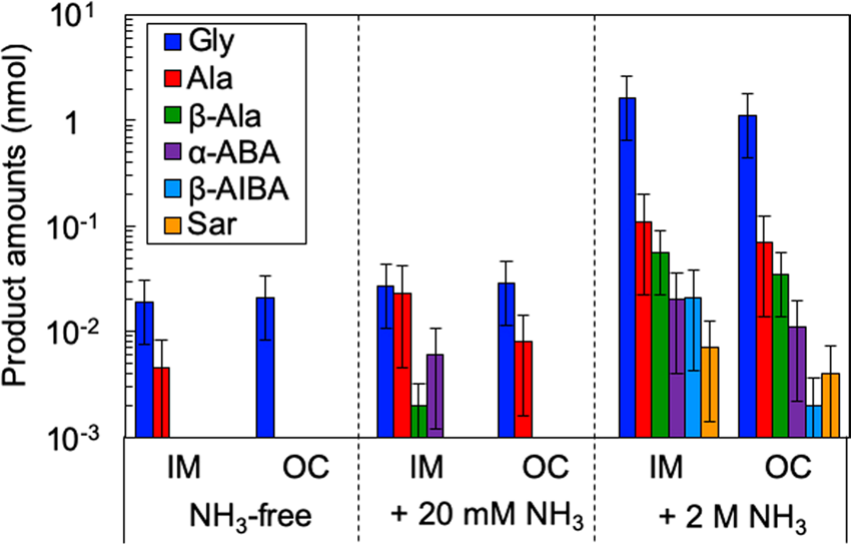
Amounts of amino acids (glycine, Gly; alanine, Ala; *β*-alanine, *β*-Ala; *α*-amino butyric acid, *α*-ABA; *β*-amino-iso-butyric acid, *β*-AIBA; sarcosine, Sar) produced in the iron meteorite (IM) and ordinary chondrite (OC) experiments (NH_3_-free, 20 mmol/L NH_3_ added, 2 mol/L NH_3_ added). Experiments were conducted three times for each condition and 1σ errors are shown.

^13^C-labeled amino acids (glycine and alanine) were synthesized in the shock-recovery experiment from the IM analogue, H_2_O, bicarbonate and N_2_ (Fig. 1). Glycine was also formed using the OC analogue in place of the IM analogue (Fig. S1). The molar conversion rates from gaseous nitrogen to amino acids were ~1 × 10^−4^% in both experiments. Six kinds of amino acids (glycine, alanine, *β*-alanine, *α*-amino butyric acid, *β*-amino-iso-butyric acid, and sarcosine) were formed in the IM and OC experiments containing 2 mol/L NH_3_ (Figs. 2, S2 and S3). Molar conversion rates from gaseous nitrogen N to amino acids were ~1 × 10^−3^% in both the IM and OC experiments. In the starting materials, N speciation was 87 mol% NH_3_ and 13 mol% N_2_. The conversion rates of these experiments were one order of magnitude higher than that of the NH_3_-free experiments, in which all N in the starting materials was N_2_. In the experiments containing 20 mmol/L NH_3_, in which 94 mol% of N in the starting materials was N_2_, the conversion rate was comparable to the NH_3_-free experiments.

**Figure 3:**
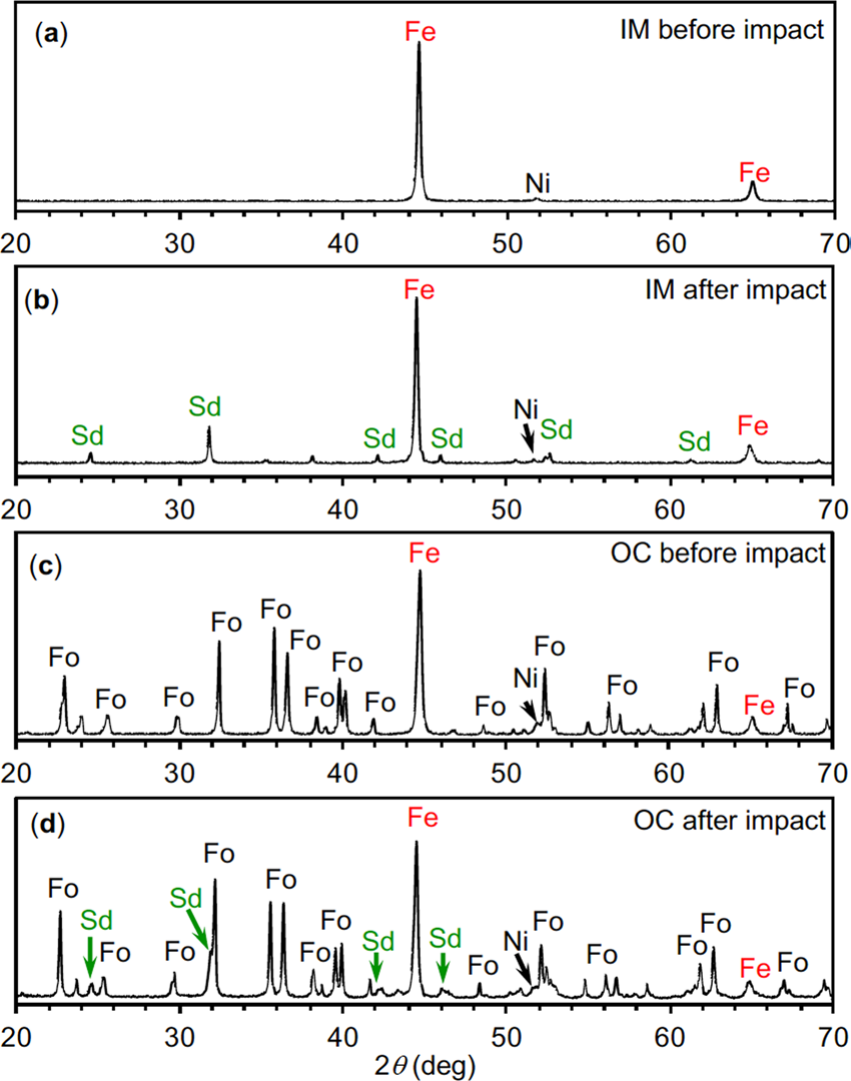
Powder X-ray diffraction profiles of solid residues after impacts. (**a**) Iron meteorite (IM) analogue before impact. (**b**) IM analogue after impact. (**c**) ordinary chondrite (OC) analogue before impact. (**d**) OC analogue after impact. Sd: siderite, Fo: forsterite.

More amino acids formed in the IM analogue experiment than in the experiment that used the OC analogue. The IM analogue contained three times more metallic Fe than the OC analogue and did not contain silicates. In the experiments using the IM analogue, metallic Fe was still the major mineral component after impact, although part of the Fe was oxidized to form FeCO_3_, i.e., siderite (Fig. 3a,b). Partial oxidation of Fe and the formation of siderite were also found in the experiments using the OC analogue (Fig. 3c,d). The effects of NH_3_ concentrations were negligible on siderite formation.

A part of NaHCO_3_ was dissolved in water and was presented as HCO_3_^−^ in the starting materials. A previous *ab initio* molecular dynamics calculation showed the formation of formic acid (HCOOH) from HCO_3_^−^ and H with metallic Fe oxidation after several picoseconds under high pressure and high temperature conditions generated by a 5 km/s meteorite impact^45^. In the present experiments, the duration of shock compression was >100 times longer than the calculation, and further HCO_3_^−^ reduction most likely formed formaldehyde (H_2_CO). Formaldehyde condenses to form larger aldehydes in alkaline solution^46^ and formation of various amino acids from aldehydes and ammonia have been well known^47,48^. In this type of amino acid synthesis, smaller amino acids form in larger yields and therefore larger amino acids would not be formed in detectable amounts in this small-scale simulation. More amino acids might have been formed if the starting martials were larger enough like natural impacts.

The formation of NH_3_ in impact-induced reactions with metallic Fe has been shown in both laboratory and numerical simulations^37,39^. In the cooling period, amino acids would be formed from aldehydes with the involvement of NH_3_ since amino acids are unstable at high temperatures, typically >300 °C (see Supplementary Information). Higher yields of simpler amino-acids are consistent with this formation via mono-carbon compounds such as formaldehyde (i.e., higher yields of glycine than alanine; Fig. 2). Similar yields of glycine in NH_3_-free experiments and 20 mM NH_3_ experiment suggest that NH_3_ formed during the impact-induced reactions worked similarly to the ammonia in the starting material. Thus, the 10^−5^ mol/L oceanic ammonia concentration suggested in a previous work may not provide a significant difference in amino acid synthesis from an NH_3_-free ocean in such a type of impact synthesis.

Abiotic syntheses of various organic compounds, including amino acids, have been successful in previous impact experiments when reduced forms of C and N were used as starting materials^17,21^. However, such reduced species were not the most likely major components of the early Earth’s atmosphere and ocean, and their availability is unclear. Formation of amino acids from non-reduced C and N sources, which were abundant on Earth during the Hadean, proposes an additional source other than the reactions associated with spark discharge (Figs. 2a and S1).

In natural meteorite/asteroid impacts, small projectiles less than 20 m tend to be broken in air and do not impact on the ground surface with hypervelocity (i.e., more than several km/sec)^49^. Projectiles larger than 100 m radius are expected to impact the ground surface with an initial velocity (i.e., mostly more than 11 km/sec) and these impactors are expected to be crashed significantly by the impact and react with terrestrial materials. Projectiles having 20 to 100 m radius have various impact velocities. Further, temperature profile of materials in an impactor is not homogeneous through the impact process; it differs significantly depending on its location in the impactor^50^. The temperature and pressure profile of the present impact would be applicable to some part of materials in the middle scale projectiles having 20 to 100 m radius.

Furthermore, the organic synthesis coupled with meteoritic Fe oxidation in impacts would be applicable to larger impactors. Larger impacts tend to be by iron-rich meteorites^51^. There was an incomplete oxidation of Fe followed by a limited formation of reactive reduced species (e.g., NH_3_, CO, H_2_, and formaldehyde) in the laboratory simulation due to limited impact conditions. In natural hypervelocity impacts, on the other hand, far greater amounts of shock heating are expected and this might have reduced more nitrogen and carbon. The organic synthesis might have been more significant, yielding a variety of amino acids in higher yields. This is supported by the higher yields and larger variations of amino acids in a higher ammonia concentration experiment that yielded six kinds of amino acids (Fig. 2).

The amounts of extraterrestrial objects accreted during the LHB period has been estimated as 4 × 10^23^ g^52^, and the contents of metallic Fe in OCs (e.g., H chondrites) are ~10 wt%^42^. Therefore, a large amount of metallic Fe was provided by impacts. In the present study, reduction of Fe, followed by the formation of reduced species, initiated the formation of amino acids. The conversion of the consumed Fe to amino acid was 2 × 10^−5^ wt% and N_2_ to amino acid was 1 × 10^−4^ atm% in the present NH_3_-free OC analogue experiment. The amount of amino acid production by the Hadean impacts would not be small, considering the huge Fe flux during the late Hadean (~4 × 10^22^ g, assuming 10 wt% of total LHB amount) and almost infinite amounts of CO_2_ and N_2_ (e.g., 10^21^ g on the modern Earth or might have been half of the modern level) in atmosphere of the Earth at that time^3,27,53^. It is difficult to precisely estimate the amounts of amino acid products generated via natural impacts with the present experiments due to technical difficulties in the demonstration of large impacts, which are expected to yield more amino acids. Alternatively, the present yield would be a baseline amount along with variation.

Extraterrestrial delivery is another source of the building blocks of life on prebiotic Earth^54–56^. Many amino acids have been detected from meteorites^57,58^. However, the amounts of the delivery remained unclear, because the composition of the building blocks of life in different carriers, particularly micrometeorites that are regarded as the largest carrier, remained unclear^52,59,60^. Further investigations regarding both endogenous formation and exogenous delivery are indispensable for understanding the availability of the building blocks of life on prebiotic Earth.

Since impact events occur locally, produced organic compounds distribute locally around the site of the impacts. This may have advantage in subsequent chemical evolution, since enrichment is one of the most significant problem in chemical evolution of polymerization^38,43,61^.

Impact-induced amino-acid formation might have been possible on Noachian Mars. The high intensity of crater distribution on Martian Noachian highland surface suggests the presence of intense impacts on Mars before 3.7 billion years ago^62,63^. A considerable amount of geological and geophysical evidence indicates a wide distribution of liquid water on Noachian Mars^63,64^ (see Supplementary Information). Atmospheric modeling combined with geological data has suggested that the major atmospheric components of Noachian Mars were CO_2_ and N_2_ with minor amounts of reduced species (see Supplementary Information). Therefore, the results of the present study further suggest that Noachian impact events synthesized abiotic organic matter including amino acids on Mars. Complex organic matter found by Mars Science Laboratory in ~3.5 Ga sediments support this implication^65^. Thus, chemical evolution might have been promoted and accomplished to the formation of monomers of catalytic biopolymers even on Noachian Mars.

## Materials and Methods

### Materials

Meteorite analogues were composed of metallic Fe (99.9 wt%, powder <45 μm in diameter; Wako), metallic Ni (99.95 wt%, sponge; Wako), and natural forsterite (Mg_2_SiO_4_) from Myanmar, which were ground and heat-treated at 450 °C for 6 h prior to use. These powders were mixed using an agate mortar and pestle. NaH^13^CO_3_ was prepared with commercial ^13^C-labeled CO_2_ (Cambridge Isotope Lab) and NaOH (Wako). Meteorite analogue material was contained in a sample container with purified water (distilled and further purified with MilliQ; 18.2 MΩ), NaH^13^CO_3_, and gaseous N (>99.995%). Details of the sample alignments are presented elsewhere^21^. All glassware for the experiments were washed and heated at 450 °C for 6 h prior to use. Sample containers were washed with water, methanol, and dichloromethane repeatedly before use.

### Shock-recovery experiments

The shock recovery experiments were conducted at the National Institute for Materials Science, Japan, using a 5 m long single stage propellant gun. Shockwaves were generated by the impacts between a 1 km/sec accelerated SUS304 disk and the sample container made of SUS304L. Detailed temperature and pressure profiles were similar to those reported in a previous study^44^.

### Sample analysis

After impact, the sample container was trimmed and washed repeatedly with water, methanol, and dichloromethane. The container was then cooled with liquid nitrogen, and holes were made as a means of accessing the sample cavity. The frozen container was immersed with water and soluble organic compounds were extracted after the ice melted. The supernatant was dried and analyzed using ultrahigh performance liquid chromatography tandem mass spectrometry (Shimadzu LCMS-8040; UHPLC/MSMS) after derivatization with AccQ-Tag reagent (Waters). Details of the UHPLC/MSMS conditions are described elsewhere^21^. The mineral composition was evaluated via a powder X-ray diffractometer (Philips) as described elsewhere^21^.

## Supplementary information

Supplementary information.

## Data Availability

All data needed to evaluate the conclusions in the paper are present in the paper and/or the Supplementary Materials. Additional data related to this paper may be requested from the authors.
