# Supplementary material for: Impact-induced amino acid formation on Hadean Earth and Noachian Mars
Source: Sci Rep. 2020 Jun 8;10:9220. doi: 10.1038/s41598-020-66112-8 (PMC7280214; doi:10.1038/s41598-020-66112-8)
Supplement: Supplementary file 1 — Supplementary information. [file 41598_2020_66112_MOESM1_ESM.pdf]

Supplementary Information

Impact-induced amino acid formation on Hadean Earth and Noachian Mars

Yuto Takeuchi[1], Yoshihiro Furukawa[1*], Takamichi Kobayashi[2], Toshimori Sekine[3,4], Naoki Terada[5], Takeshi Kakegawa[1]

1. Supplementary Discussion

1.1. Environments on Noachian Mars

A popular model for the trigger of the terrestrial heavy impacts that occurred during the end of the Hadean and the beginning of the Archean is the large-scale rearrangement of the giant planets' orbits[64]. In this scenario, the inner planets, including Mars, also experienced intense impacts by meteorites and asteroids. A high intensity of crater distribution on Martian Noachian highland surfaces indicate the presence of intense impacts on Mars before 3.7 billion years ago[62,63]. Geological and geophysical evidence, including a wide distribution of phyllosilicates and valley networks, indicate the presence of liquid water on Noachian Mars[63,64]. The size of the water body remains uncertain, but the stratigraphy, geomorphology, and topography records indicate the presence of a vast ocean that covered the northern hemisphere around 3.7 billion years ago[65,66]. The redox state of minerals in Martian meteorites (Shergottites) indicates that the redox state of the early Martian mantle was represented by quartz-fayalite-magnetite (QFM) 4 to QFM–1, which is more reduced than of that of the terrestrial mantle[67]. This indicates that volcanic gases might have provided reduced species such as CO and CH_4 in addition to CO_2. The actual atmospheric composition was also controlled by the atmospheric species escaping to space, which preferentially removed lighter species such as hydrogen, thereby oxidizing the surface environment over geological timescales[68]. Batalha et al.[69] estimated that plausible atmospheric components of Noachian Mars were CO_2 and N_2 with minor amounts of CO and H_2, supposing a continuous degassing with a redox state represented by iron-wüstite (IW) –3. The presence of carbonates in Noachian sediments also support a CO_2-dominated atmosphere[70]. These geological conditions are not too different from the conditions on Hadean Earth. Thus, impact-induced amino-acid formation would be applicable to Noachian Mars as well. Some geological records suggest that Martian aqueous environments after the Noachian were sometimes more oxidized, and within which hematite occurred. In such oxidized environments, the formation of organic compounds might have been more difficult than on Noachian Mars[71].

1.2. Potential synthesis reactions of amino acids

The formation of NH_3 in impact-induced reactions with Fe, H_2O, and N_2 have been found in previous experimental and theoretical simulations[37,39]. *Ab-initio* calculation has indicated that HCO_3^- and CO_2 are reduced to form formic acid (HCOOH) in an incipient reaction during shock compression[45]. Further reduction most likely produced formaldehyde (H_2CO), since the duration of the shock compression was far longer in the present experiments.

The most famous synthetic route of amino acids in prebiotic reactions is the Strecker reaction, in which hydrolysis of amino nitrile (formed by the reaction between formaldehyde, hydrogen cyanide (HCN), and NH_3) results in the formation of α-amino acids[70]. However, in the present study, the Strecker reaction might not have been responsible for the amino-acid synthesis since the samples were not hydrolyzed and β- and γ-amino acids formed simultaneously.

Formaldehyde reacts with other formaldehyde to form glycolaldehyde and longer aldehydes[46]. Formation of glycine, α-alanine, and β-alanine by the amination of aldehydes have been shown in previous studies[47]. This reaction most likely produced amino acids in the present impact-induced reactions, since this reaction does not require hydrolysis and can explain the simultaneous formation of α- and β-amino acids.

Supplementary References

64. Tsiganis, K., Gomes, R., Morbidelli, A., Levison, H. F., Origin of the orbital architecture of the giant planets of the Solar System. *Nature* **435**, 459–461 (2005).

65. Fairén, A. G. *et al.*, Episodic flood inundations of the northern plains of Mars. *Icarus* **165**, 53–67 (2003).

66. Di Achille, G., Hynek, B. M., Ancient ocean on Mars supported by global distribution of deltas and valleys. *Nat. Geosci.* **3**, 459–463 (2010).

67. Schmidt, M. E., Schrader, C. M., McCoy, T. J., The primary fO_2 of basalts examined by the Spirit rover in Gusev crater, Mars: Evidence for multiple redox states in the martian interior. *Earth Planet. Sci. Lett.* **384**, 198–208 (2013).

68. Lammer, H. *et al.*, Loss of water from Mars: implications for the oxidation of the soil. *Icarus* **165**, 9-25 (2003).

69. Batalha, N., Domagal-Goldman, S. D., Ramirez, R., Kasting, J. F., Testing the early Mars H_2-CO_2 greenhouse hypothesis with a 1-D photochemical model. *Icarus* **258**, 337–349 (2015).

70. Ehlmann, B. L. *et al.*, Orbital identification of carbonate-bearing rocks on mars. *Science* **322**, 1828–1832 (2008).

71. Squyres, S. W. *et al.*, In situ evidence for an ancient aqueous environment at Meridiani Planum, Mars. *Science* **306**, 1709–1714 (2004).

72. Kouznetsov, V. V., Galvis, C. E. P., Strecker reaction and α-amino nitriles: Recent advances in their chemistry, synthesis, and biological properties. *Tetrahedron* **74**, 773–810 (2018).

Supplementary Table

Table S1. Amounts of starting materials, impact velocities, and products.

Type			NH_3-free		20 mM NH_3		2 M NH_3	
			IM	OC	IM	OC	IM	OC
Starting materials	Fe	(mg)	300	100	300	100	300	100
	Ni	(mg)	10	10	10	10	10	10
	Mg_2SiO_4	(mg)	0	200	0	200	0	200
	$NaH^{13}CO_3$	(mg)	200	200	200	200	200	200
	NH_3	(mmol)	0	0	0.8	0.8	80	80
	H_2O	(mg)	130	130	130	130	130	130
	N_2 (gas)	(μmol)	20	20	20	20	20	20
Impact velocity	(km/sec)		0.87	0.86	0.88	0.87	0.87	0.85
Product amino acids	Gly	(nmol)	0.019	0.021	0.027	0.029	1.6	1.1
	Ala	(nmol)	0.005	-	0.023	0.008	0.11	0.069
	β-Ala	(nmol)	-	-	0.002	-	0.056	0.035
	α-ABA	(nmol)	-	-	0.006	-	0.02	0.011
	β-AIBA	(nmol)	-	-	-	-	0.021	0.002
	Sar	(nmol)	-	-	-	-	0.007	0.004

OC: ordinary chondrite analogue; IM: iron meteorite analogue; α-ABA: α-amino butyric acid; β-AIBA: β-amino-iso-butyric acid. Amounts of products and impact velocities are shown as average values based on three experiments. The variation of impact velocity is $\pm$ 0.02 km/sec. 1σ errors of the product amounts are shown in Fig. 2.

Supplementary Figures

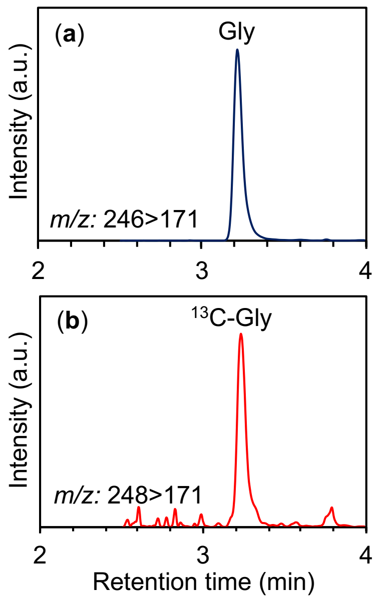

Figure S1. Multiple reaction monitoring (MRM) chromatogram of derivatized standards and the derivatized product of the NH_3-free experiment with ordinary chondrite (OC) analogue. (**a**) ^{12}C-glycine (Gly) (*m/z* = 246 > 171). (**b**) Product, ^{13}C-glycine (^{13}C-Gly) (*m/z* = 248 > 171).

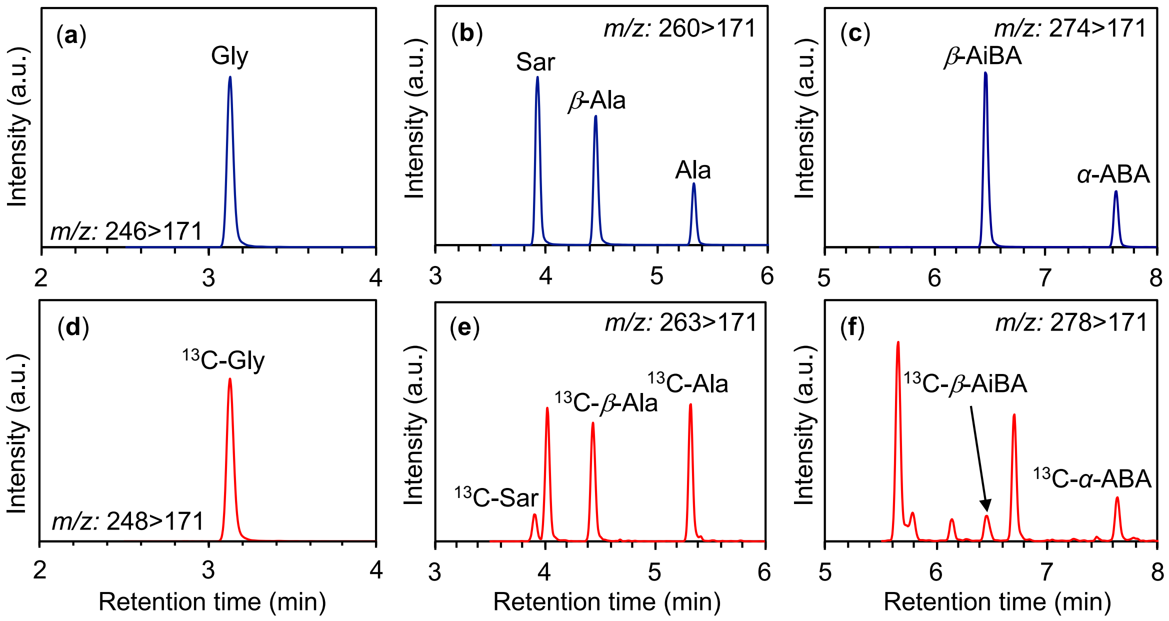

Figure S2. Multiple reaction monitoring (MRM) chromatogram of derivatized standards and the derivatized product of the 2 M NH_3 experiment using the iron meteorite (IM) analogue. (**a**) ^{12}C-glycine (Gly) (*m/z* = 246 > 171). (**b**) ^{12}C-alanine (Ala), ^{12}C-β-alanine (β-Ala), and sarcosine (Sar) (*m/z* = 260 > 171). (**c**) ^{12}C -β-amino-iso-butyric acid (^{12}C-β-AiBA) and ^{12}C-α-amino butyric acid (^{12}C-α-ABA) (*m/z* = 274 > 171). (**d**) Product, ^{13}C-glycine (^{13}C-Gly) (*m/z* = 248 > 171). (**e**) Products, ^{13}C-sarcosine (^{13}C-Sar), ^{13}C-β-alanine (^{13}C-β-Ala), and ^{13}C-alanine (^{13}C-Ala) (*m/z* = 263 > 171). (**f**) Products, ^{13}C -β-amino-iso-butyric acid (^{13}C-β-AiBA) and ^{13}C-α-amino butyric acid (^{13}C-α-ABA) (*m/z* = 278 > 171).

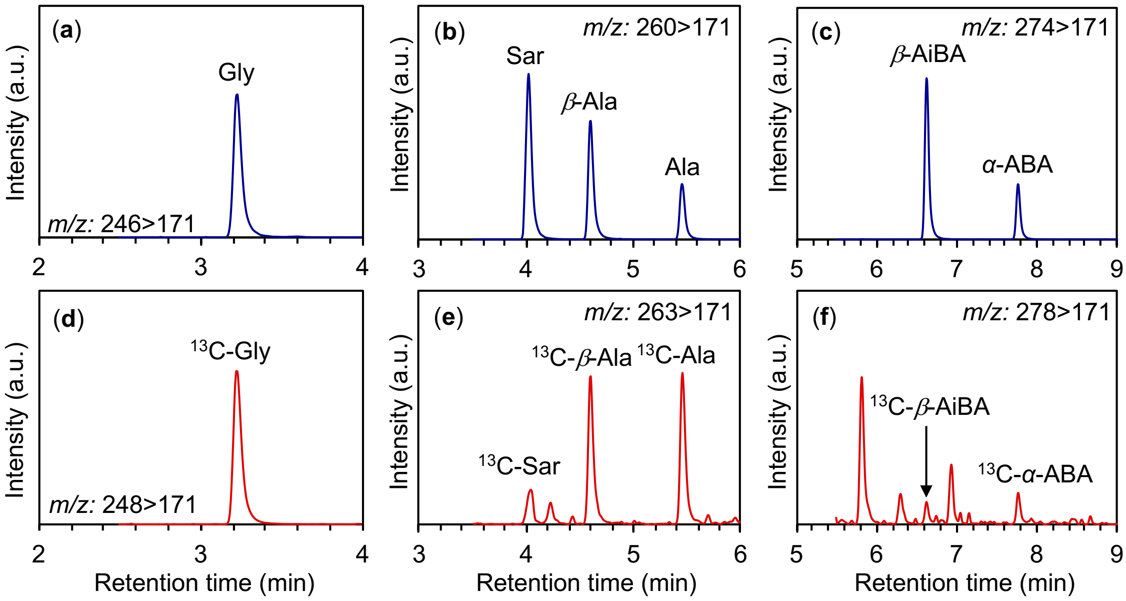

Figure S3. Multiple reaction monitoring (MRM) chromatogram of derivatized standards and the derivatized product of the 2 M NH_3 experiment using the ordinary chondrite (OC) analogue. (**a**) ^{12}C-glycine (Gly) (*m/z* = 246 > 171). (**b**) ^{12}C-alanine (Ala), ^{12}C-β-alanine (β-Ala), and sarcosine (Sar) (*m/z* = 260 > 171). (**c**) ^{12}C-β-amino-iso-butyric acid (β-AiBA) and ^{12}C-α-amino butyric acid (α-ABA) (*m/z* = 274 > 171). (**d**) Product, ^{13}C-glycine (^{13}C-Gly) (*m/z* = 248 > 171. (**e**) Products, ^{13}C-sarcosine (^{13}C-Sar), ^{13}C-β-alanine (^{13}C-β-Ala), and ^{13}C-alanine (^{13}C-Ala) (*m/z* = 263 > 171). (**f**) Products, ^{13}C -β-amino-iso-butyric acid (^{13}C-β-AiBA) and ^{13}C-α-amino butyric acid (^{13}C-α-ABA) (*m/z* = 278 > 171).
